# Psychotic Symptoms in Kenya – Prevalence, Risk Factors, and Relationship with Common Mental Disorders

**DOI:** 10.3390/ijerph9051748

**Published:** 2012-05-07

**Authors:** Rachel Jenkins, Frank Njenga, Marx Okonji, Pius Kigamwa, Makheti Baraza, James Ayuyo, Nicola Singleton, Sally McManus, David Kiima

**Affiliations:** 1 Director, WHO Collaborating Centre (Mental Health), Institute of Psychiatry, Kings College London, PO 35, De Crespigny Park, London SE5 8AF, UK; 2 Consultant Psychiatrist, Upper Hill Medical Centre, Nairobi, Kenya; Email: fnjenga@africaonline.co.ke; 3 Consultant Psychiatrist, The Nairobi Hospital, Nairobi, Kenya; Email: marxokonji@wananchi.com; 4 Department of Psychiatry, University of Nairobi, Nairobi, Kenya; Email: pkigamwa@africaonline.co.ke; 5 Ministry of Health, Survey Department, Nairobi, Kenya; 6 Mildmay International, Kisumu, Kenya; Email: jayuyo@mildmay.or.ke; 7 Director of Policy & Research, UK Drug Policy Commission, London, UK; Email: NSingleton@ukdpc.org.uk; 8 Research Director, National Centre for Social Research (NatCen), London, UK; Email: s.mcmanus@natcen.ac.uk; 9 Director of Mental Health, Ministry of Health, Kenya; Email: dmkiima@gmail.com

**Keywords:** epidemiology, Kenya, psychosis, development

## Abstract

There have been few epidemiological surveys to establish prevalence and associated risk factors of psychosis in Sub-Saharan Africa. This paper reports a population- based epidemiological survey in rural Kenya of the prevalence of psychotic symptoms and their relationship with demographic, socio-economic and other risk factors. A random sample of 2% of all adults living in Maseno, Kisumu District of Nyanza province, Kenya (50,000 population) were studied, aiming for a sample size of 1,000 people. The psychosis screening questionnaire was used to assess the prevalence of psychotic symptoms in the preceding twelve months. The response rate was 87.6%. The prevalence of single psychotic symptoms in rural Kenya was 8% of the adult population, but only 0.6% had two symptoms and none had three or more psychotic symptoms in this sample size. Psychotic symptoms were evenly distributed across this relatively poor rural population and were significantly associated with presence of common mental disorders, and to a lesser extent with poor physical health and housing type. We conclude that single psychotic symptoms are relatively common in rural Kenya and rates are elevated in those with CMD, poor physical health and poor housing.

## 1. Introduction

This was a collaborative project with the ministries of health in Tanzania and Kenya, funded by the UK Department for International Development and aimed at conducting detailed situation appraisal, policy development and implementation in both countries [1,2]. As part of the situation appraisal, two epidemiological surveys were conducted in urban Tanzania and rural Kenya, aiming to establish the prevalence of common mental disorders, psychosis and substance use and misuse in both settings. The results of the Tanzanian survey have been reported elsewhere [3,4,5]. This paper reports the results of the Kenyan survey in relation to the prevalence of psychotic symptoms in rural Kenya and their relationship with demographic, socio-economic and other risk factors. The results for common mental disorders (CMD) and substance use will be reported elsewhere.

Surveys of psychosis have been conducted using clinician administered instruments [6] which can establish both psychotic symptom severity and diagnostic category; using family reports [7], and using systematic assessment of psychotic symptoms by detailed interviews administered by non-medical interviewers, leading to enumeration of symptom frequency and severity, and estimate of probable psychosis [8,9].

## 2. Methods

### 2.1. Site

The study sample frame was the 50,000 population living in Maseno, in Kisumu District of Nyanza Province, Kenya. This is a poor rural largely agricultural area of western Kenya, on the edge of Lake Victoria, the majority tribe being Luo. The province has been subject to political, economic and ethnic tensions. 

### 2.2. Sample

All households in Maseno had been enumerated in the 2000 Government of Kenya Census. One in 50 households of 50,000 population were sampled at random to give a sample size of 1000 households. Each of the 1,000 sampled households were visited, all members recorded in order of age, and one eligible person aged 16–65 selected at random from each household for interview. The age cut off of 65 was applied as at that stage we did not have experience of applying the instrument to people aged over 65, some of whom would have cognitive deficits. The final sample size was 876.

### 2.3. Implementation of the Survey

Efforts were made to use local capacity. Pencil and paper administration of the survey was coordinated by the Ministry of Health section for surveys. Interviews were conducted by lay volunteer community health workers, with no formal mental health training, linked to primary care centres in Maseno. Interviewers received a brief orientation to the instrument and were trained in its use. Responses were recorded verbatim, scored and entered into an SPSS database. The interviewers were supervised by a public health nurse working in Chulaimbo Rural Health Training Centre in Maseno. Informed consent was obtained from all respondents. Interviews were conducted in English where possible or Luo if necessary, as required by the respondent.

### 2.4. Assessments

The overall survey comprised sections on demographic and socio-economic factors, common mental disorders, psychosis, substance use, life events and social supports.

### 2.5. Demographic and Socioeconomic Factors

Demographic information collected included sex, age, marital status and ethnicity, as well as household status (*i.e.*, whether the respondent was head of household, spouse or other). Socioeconomic information included employment status, education attainment, housing tenure (*i.e.*, whether the home was owned or rented) and type of accommodation (whether the respondent lived in a ‘permanent home (brick, corrugated iron), “semi-permanent” (mud) or “temporary” home (leaves, branches, sticks). 

### 2.6. Psychosis

The Psychosis Screening Questionnaire (PSQ) was used to assess the presence of psychotic symptoms in the preceding twelve months [10]. The PSQ was developed for use by lay interviewers and uses five probe stem questions, with one or two follow up questions for those that answered yes to the probe stem question, to determine recent experience of hypomania, thought interference, paranoia, a feeling that something strange is taking place that is hard to explain, and hallucinations. The informant must have answered yes to all questions within a symptom category in order to screen positive on that item. 

In the standard use of the PSQ, informants are not asked to continue the psychosis screening questionnaire once they have answered positively to one item, because a positive screen would route the informant to a more detailed clinical assessment. However, in this study, which did not conduct clinical assessments, informants were asked all of the stem questions, regardless of their response to earlier ones. 

### 2.7. Common Mental Disorders

The Clinical Interview Schedule–Revised (CIS-R) is a gold standard instrument for assessing current common mental disorders by lay interviewers in community settings [11]. It has been widely used in relatively rich countries such as Britain, and also increasingly in low income countries, including the neighbouring country of Tanzania [4,12,13]. 

The CIS-R provides ICD diagnoses of depressive episode (mild, moderate or severe), obsessive compulsive disorder, panic disorder, phobic disorder, generalised anxiety disorder and mixed anxiety/depressive disorder. It does not assess PTSD. These diagnoses were the basis for an overall category of common mental disorder (otherwise non-psychotic disorder or neurosis). 

### 2.8. Analysis

The raw data were weighted. The weights were calculated to take account of selection bias due to household size and to correct for the oversampling of Head of Household (HoH) and spouse, by weighting down those with a status of HoH/spouse.

Data was analysed using SPSS software for Windows Version 15 [14]. Prevalence rates were calculated for psychotic symptoms, and for CMD, and prevalence rates of psychotic symptoms were compared across demographic and socioeconomic characteristics. The sample size was not large enough to analyse diagnostic categories separately Odd ratios (OR) with 95% confidence intervals (CI) were calculated to determine significant associations with the prevalence of any psychotic symptom within each area. Endorsement of at least one psychotic symptom was then examined as a dependent variable in a multiple logistic regression for each area. All significant factors identified in the bi-variate analyses were entered into the final models along with all demographic variables.

### 2.9. Ethics Approval

Approval was granted by Mathari National Mental Hospital, Ministry of Health, Kenya, and Maudsley (SLaM), National Health Service (NHS) Foundation Trust.

## 3. Results and Discussion

The response rate was 87.6%. The prevalence of at least one psychotic symptom in this group was 8.1% with only 0.6% having two symptoms and none having three or more symptoms (Table 1). Thought insertions and paranoia were the commonest psychotic symptoms at 3.6 and 3.5%. The prevalence of CMD in this sample was 10.8%, largely comprising mixed anxiety depression (6.1%), panic disorder (2.6%), generalised anxiety disorder (1.6%) and depressive episodes (0.7%) (see Table 1). Table 2 shows that the presence of psychotic symptoms was highly associated with the presence of CMD, but was not associated with demographic or socio-demographic variables in this sample (Table 3), although type of home and presence of poor physical health were associated at the P < 0.1 level of significance. 

**Table 1:** Prevalence of psychotic symptoms and CMD in a community based sample in Maseno.

	n	%	Standard deviation
	n = 876		
**One or more psychotic symptom ^a^**	59	8.1	0.28
**PSQ score (number of symptoms present)**			0.30
0	817	91.9	
1	54	7.6	
2	5	0.6	
3+	0	0.0	
**Type of symptom present**			
Thought insertions	9	3.4	0.18
Paranoia	32	3.3	0.18
Strange experiences	14	1.5	0.12
Hallucinations	6	0.7	0.09
Mania	3	0.1	0.04
**Any CMD ^b^**	83	10.8	0.31
**Specific CMDs**			
Mixed anxiety and depression	48	6.1	0.24
Panic disorder	17	2.6	0.16
Generalised anxiety disorder	14	1.6	0.13
Depressive episode	9	0.7	0.08
Phobic disorder	3	0.3	0.05
Obsessive compulsive disorder	2	0.2	0.04

^a^ Psychotic symptoms in the preceding year as measured by the five domains of the Psychosis Screening Questionnaire (PSQ); ^b^ Any common mental disorder (CMD) and specific common mental disorder in the past seven days as measured by the Clinical Interview Schedule–Revised (CIS-R).

**Table 2:** Prevalence and unadjusted odds ratio for psychotic symptoms by presence of CMD.

	Prevalence of PSQ symptoms %	Unadjusted odds ratio	CI (95%)
No CMD	4	1	
Any CMD	43.2	**17.58 ^a^**	**(5.23, 59.12)**

^a^ p = 0.000. Common mental disorder (CMD) Psychosis Screening Questionnaire (PSQ)

**Table 3:** Prevalence rates and unadjusted odds ratios of any psychotic symptoms in relation to background factors.

	n	Prevalence of PSQ symptoms %	Unadjusted odds ratio	CI (95%)
**Sex**				
Male	286	9.4	1.0	
Female	563	6.9	0.73	(0.43, 1.25)
**Age group (years)**				
16–29	304	6.6	1.0	
30–44	269	9.2	1.4	(0.48, 4.10)
45–64	293	9.8	1.5	(0.65, 3.52)
**Marital status**				
Married/cohabitating	584	8.4	1.0	
Single	171	10.5	1.3	(0.60, 2.63)
Widowed/divorced/separated	58	5.2	0.7	(0.31, 1.65)
**Relationship to head of household**				
Head	311	6.4	1.0	
Spouse/partner	223	5.4	0.9	(0.49, 1.52)
Son/daughter/other	256	10.9	1.8	(0.59, 5.37)
**Education level**				
None	112	12.0	1.0	
Primary	558	6.2	0.48	(0.15, 1.53)
Secondary	146	15.6	1.33	(0.55, 3.20)
Post secondary	43	3.8	0.26	(0.03, 2.65)
**Employment status**				
None	115	10.4	1.0	
Farmer	488	7.4	0.7	(0.22, 2.26)
Casual/wage worker	88	11.4	1.2	(0.14, 10.01)
Trade/business	88	10.2	1.0	(0.34, 3.07)
**Type of home ^a^**				
Permanent structure	169	12.7	1.0	
Semi-permanent	466	8.8	0.7	(0.20, 2.26)
Temporary	233	4.2	**0.3**	**(0.11, 0.96)**
**Poor health ^b^**				
No	735	6.9	1.0	
Yes	105	20.0	2.50	(0.90, 12.16)

^a^ p = 0.07; ^b^ p = 0.09. Psychosis Screening Questionnaire (PSQ)

## 4. Discussion

There has been a paucity of systematic community surveys of psychosis using standardised instruments in Africa [15]. This rural study in Kenya using the PSQ has found an annual psychotic symptom rate of 8.1%. No relationship was found between prevalence of psychotic symptoms and those background variables studied. 

The annual prevalence of one or more psychotic symptoms reported here was somewhat higher than findings from Ethiopia, where a prevalence of 6.0% was observed for psychotic symptoms using the Self-Reporting Questionnaire in rural Ethiopia [16], and in Tanzania using the PSQ where an annual prevalence of psychotic symptoms was 3.9% in an urban sample. Although population based surveys in the United States [17], the Netherlands [18], and New Zealand [19], have found somewhat higher prevalence rates for psychotic symptoms (28%, 17.5% and 20.1% respectively) in Britain, where the PSQ was used, the prevalence of psychotic symptoms was 5.5%, a figure more comparable to the current results [20]. 

The PSQ is an instrument to screen for psychotic symptoms and does not of itself confirm the presence of psychosis, and we would expect rates of one or more psychotic symptoms to be much higher than rates of three or more psychotic symptoms. Studies attempting to estimate rates of actual psychosis have been conducted in Ethiopia, Mozambique and Zanzibar. In Ethiopia, past urban month rates of combined schizophrenia and schizoaffective disorder were 0.7% using the CIDI [6], and psychotic illness was 0.3% based on clinical psychiatric interview [21]. The Mozambique study used key informants (the first person found in the randomly selected household able to answer on behalf of others) to identify disordered behaviour via vignette. The authors found higher lifetime prevalence of psychoses (4.4%) in the poorer rural area compared to 1.6% in Maputo city [7]. Using a similar methodology in Zanzibar, the rate of chronic psychosis was found to be 2.6/1000 and acute psychotic episodes 0.6/1000 [22]. 

The association between psychotic symptoms and CMD has been previously reported in the British national surveys [20], and in Tanzania [3]. Unlike most other studies–but similar to the recent Tanzania study [3]–age, gender and marital status were not found to be significantly associated with psychotic symptoms. 

The current paper also investigated household status, housing type, education, income and ethnicity, given their previously reported associations with psychotic symptoms in Britain [23], but none of these relationships was significant at the p = 0.5 level after adjustment for other variables, although there was an association with housing type (significant at the 0.9 level) and with presence of physical illness (significant at the 0.7 level). The association with physical illness has been found more strongly elsewhere [24]. It may be that a larger sample size is needed to find socio-demographic associations, especially as this was a relatively homogeneous population in terms of the above variables.

The strengths of this study are the relatively good response rate, the use of a random sample of households, the use of instruments which provide comprehensive assessments of common mental disorders, and a systematic approach to screening for psychosis. Several limitations should be noted. While the sampling frame was well defined and based on the 2000 Kenya census which had enumerated all dwellings, adequate supervision of the implementation of the survey was difficult for financial and logistical reasons. Over-representation of heads of household indicates that the random sampling of individuals within each household was inadequately implemented. 

The PSQ was not originally designed for Sub-Saharan Africa. It was therefore carefully scrutinised by local clinicians for content validity within the local cultural context, but was not tested against a gold standard interview. As the prevalence of psychotic symptoms in general population samples is commonly low, the overall sample size was not large enough to yield a great number of people reporting past-year psychotic symptoms, and therefore the power to detect associations was limited. We did not arrange to confirm probable psychosis with a follow-up clinical interview by trained psychiatrists, due to the high opportunity cost of such an exercise in a low income country with few psychiatrists (there is only one psychiatrist in Nyanza Province for around 5 million population). As always, the potential for measurement error when using screening instruments should be acknowledged, given self-reported experiences may be subject to recall or social desirability response bias. In addition the lay volunteer community health workers were not experienced survey interviewers. We do not know whether some of the psychotic symptoms here were alcohol or drug related, as the data on substance abuse was insufficiently complete to analyse. Finally, the current findings are specific to Maseno division in Nyanza Province and are not necessarily applicable to other parts of Kenya, particularly urban areas.

## 5. Conclusions

Single psychotic symptoms in rural Kenya are relatively common (8%) in the adult population, but with only 0.6% having two symptoms, and in this sample size none were found with three or more symptoms. Symptoms are evenly distributed across this relatively poor rural population, and are significantly associated with CMD, and to a lesser extent with poor physical health and housing type. Health and social care systems in Kenya should take psychotic symptoms into account when planning for both physical and mental disorders.

## References

[1] Mbatia J., Jenkins R. (2010). Mental health policy in Tanzania. Psychiatr. Serv..

[2] Kiima D., Jenkins R. (2010). Mental health policy in Kenya - an integrated approach to scaling up equitable care for poor populations. Int. J. Ment. Health Syst..

[3] Jenkins R., Mbatia J., Singleton N., White B. (2010). Prevalence of psychotic symptoms and their risk factors in urban Tanzania. Int. J. Environ. Res. Public Health.

[4] Jenkins R., Mbatia J., Singleton N., White B. (2010). Prevalence of common mental disorders and their risk factors in urban Tanzania. Int. J. Environ. Res. Public Health.

[5] Mbatia J., Jenkins R., Singleton N., White B. (2009). Prevalence of alcohol consumption and hazardous drinking, tobacco and drug use in urban Tanzania, and their associated risk factors. Int. J. Environ. Res. Public Health.

[6] Kebede D., Alem A. (1999). Major mental disorders in Addis Ababa, Ethiopia: I. Schizophrenia, schizoaffective and cognitive disorders. Acta Psychiatr. Scand..

[7] Patel V., Simbine A.P.F., Soares I.C., Wiess H.A., Wheeler E. (2007). Prevalence of severe mental disorder and neurological disorders in Mozambique: a population-based survey. Lancet.

[8] Samele C., van Os J., McKenzie K., Wright A., Gilvarry C., Manley C., Tattan T., Murray R. (2001). Does socioeconomic status predict course and outcome in patients with psychosis?. Soc. Psychiatry Psychiatr. Epidemiol..

[9] Lewis G., Bebbington P., Brugha T., Farrell M., Gill B., Jenkins R., Meltzer H. (1998). Socioeconomic status, standard of living, and neurotic disorder. Lancet.

[10] Bebbington P.E., Nayani T. (1995). The psychosis screening questionnaire. Int. J. Methods Psychiatr. Res..

[11] Lewis G., Pelosi A.J., Araya R.C., Dunn G. (1992). Measuring psychiatric disorder in the community: A standardised assessment for use by lay interviewers. Psychol. Med..

[12] Ngoma M.C., Prince M., Mann A. (2003). Common mental disorders among those attending primary health clinics and traditional healers in urban Tanzania. Br. J. Psychiatry.

[13] Patel V., Kirkwood B.R., Pednekar S., Weiss H., Mabey D. (2006). Risk factors for common mental disorders in women: Population-based longitudinal study. Br. J. Psychiatry.

[14] SPSS Inc. (2006). Statistical Package for Social Sciences, Windows 15 ed..

[15] (2001). Neurological, Psychiatric, and Developmental Disorders: Meeting the Challenge in the Developing World.

[16] Tafari S., Aboud F.E., Larson C.P. (1991). Determinants of mental illness in a rural Ethiopian adult population. Soc. Sci. Med..

[17] Kendler K.S., Gallagher T.J., Abelson J.M., Kessler R.C. (1996). Lifetime prevalence, demographic risk factors, and diagnostic validity of nonaffective psychosis as assessed in a US community sample. Arch. Gen. Psychiatry.

[18] van Os J., Hanssen M., Bijl R. V., Ravelli A. (2000). Strauss (1969) revisited: a psychosis continuum in the general population?. Schizophr. Res..

[19] Poulton R., Caspi A., Moffitt T.E., Cannon M., Murray R., Harrington H. (2000). Children’s self-reported psychotic symptoms and adult schizophreniform disorder: A 15-year longitudinal study. Arch. Gen. Psychiatry.

[20] Johns L.C., Cannon M., Singleton N., Murray R.M., Farrell M., Brugha T., Bebbington P., Jenkins R., Meltzer H. (2004). Prevalence and correlates of self-reported psychotic symptoms in the British population. Br. J. Psychiatry.

[21] Giel R., van Luijk J.N. (1969). Psychiatric morbidity in a small Ethiopian town. Br. J. Psychiatry.

[22] Bondestam S., Garssen J., Abdulwakil A. (1990). Prevalence and treatment of mental disorders and epilepsy in Zanzibar. Acta Psychiatr. Scand..

[23] Wiles N.J., Zammit S., Bebbington P., Singleton N., Meltzer H., Lewis G. (2006). Self-reported psychotic symptoms in the general population: Results from the longitudinal study of the British National Psychiatric Morbidity Survey. Br. J. Psychiatry.

[24] Leucht S., Burkard T., Henderson J., Maj M., Sartorius N. (2007). Physical illness and schizophrenia: a review of the literature. Acta Psychiatr. Scand..

